# P02.162. Parental interest in integrative care for children with attentional concerns

**DOI:** 10.1186/1472-6882-12-S1-P218

**Published:** 2012-06-12

**Authors:** K Kemper, B Banasiewicz

**Affiliations:** 1 Wake Forest University Health Sciences, Winston-Salem, USA

## Purpose

Many families seek natural therapies for children. We describe what families want when they seek consultation for natural therapies for children with ADHD.

## Methods

For new patients seen in an integrative pediatric clinic between 1/2010 and 6/2011, we reviewed intake forms, physician reports, and laboratory studies.

## Results

Of the 75 new patients, 23 (31%) families had concerns about ADHD. Of these, 70% were male; the average age was 11.2 ± 3.1 years; and 80% also received care from specialists. Eleven patients (48%) were taking a prescription medication, but only 3 (13%) were taking medicine for ADHD; dietary supplements were taken by 12 patients (52%). Most families were interested in health promotion information about diet (87%), exercise (78%), stress management (74%), and sleep (74%). Of 11 patients tested, 82% had low ferritin. Recommendations focused on health promotion (100%), dietary supplements such as multivitamins/minerals (71%) and omega-3 fatty acids (82%), and specialist referrals (30%).

## Conclusion

Families seeking natural therapies for children with ADHD have needs for health promotion and care coordination that are well addressed using tools and skills already present in the medical home.

